# Typically inhibiting USP14 promotes autophagy in M1-like macrophages and alleviates CLP-induced sepsis

**DOI:** 10.1038/s41419-020-02898-9

**Published:** 2020-08-20

**Authors:** Fang Xu, Yuxiang Ma, Wei Huang, Jian Gao, Mengmeng Guo, Jianxin Li, Lingdong Kong, Guang Liang, Ronghui Du, Qiang Xu, Xudong Wu

**Affiliations:** 1 State Key Laboratory of Pharmaceutical Biotechnology, Nanjing Drum Tower Hospital, School of Life Sciences, Nanjing University, 163 Xianlin Avenue, Nanjing, 210023 China; 2 State Key Laboratory of Analytical Chemistry for Life Science, School of Chemistry and Chemical Engineering, Nanjing University, 163 Xianlin Avenue, Nanjing, 210023 China; 3 Chemical Biology Research Center, School of Pharmaceutical Science, Wenzhou Medical University, Wenzhou Zhejiang, 325035 China; 4 Jiangsu Key Laboratory of Molecular Medicine, Medical School of Nanjing University, 22 Hankou Road, Nanjing, 210093 China

**Keywords:** Mechanisms of disease, Pharmacology

## Abstract

Macrophages, with diverse functions and variable phenotypes, are considered as an important executor of inflammatory diseases. And it has been proved that autophagy is deeply connected with the development of inflammation, while the exact regulatory mechanism still remains unclear, and the application of autophagy regulators in anti-inflammation needs to be further confirmed. Here, we firstly verified that neochromine S5 (hereinafter referred to as S5) significantly inhibited M1-like macrophage polarization with decrease of the proinflammatory cytokines and downregulation of NF-κB and STAT1 signals. Then, in vivo experiments demonstrated S5 improved cecal ligation and puncture (CLP)-induced sepsis specially based on the regulation of M1-like macrophages. Mechanistic studies indicated that S5 treatment dramatically upregulated cellular autophagy in M1-like macrophage. Furthermore, by multiple methods, S5 was revealed to directly bind with ubiquitin-specific proteases 14 (USP14) at Ser404, Phe405, and Cys414 by hydrogen bond to inhibit its deubiquitinating activity, and block USP14–TRAF6 (TNF receptor associated factor 6) interaction, subsequently promoting ubiquitination of Beclin1, interrupting Beclin1–Bcl2 interaction, and accumulating the autophagosome in macrophages, which finally resulted in the blockade of M1-like macrophage polarization. Animal experiments also confirmed the protection of S5 in CLP mice was dependent on activation of macrophage autophagy. What’s more, as a novel USP14 inhibitor, S5 exhibited higher efficiency and safety than IU1, the known USP14 inhibitor. Therefore, this study has demonstrated that typically inhibiting USP14 promotes autophagy in M1-like macrophages and alleviates CLP-induced sepsis. Moreover, we provide a new candidate compound, S5, for sensitizing autophagy to interfere with the macrophage inflammation.

## Introduction

Inflammation is a biological response to harmful stimuli, such as pathogens, damaged cells, or irradiation^1^. It is a protective attempt by the organism to remove injurious stimuli and to initiate the healing process^2^. However, if inflammation get out of control and excessive stimulated, which will promote systemic inflammatory diseases, such as sepsis^3^. Macrophages are an essential component of innate immunity, and play a central role in inflammation and host defense^4,5^. Macrophages are characterized by diversity and plasticity, including classically activated M1-like macrophages (stimulated by TLR ligands and IFN-γ) and alternatively activated M2-like macrophages (stimulated by IL-4 and/or IL-13)^6,7^, or other status between these two extremely phenotype^8^. M1-like macrophages are proinflammatory phenotype and express high-level proinflammatory cytokines, such as IL-1β, IL-6, TNF-α, and reactive nitrogen, and have a central role in host defense against infection. Excessively sustained activation of M1-like macrophages triggers severe inflammatory response. M1-like macrophages can be regulated as target for the therapy of various immune diseases, and inhibiting M1-like macrophage polarization has been applied in the clinical practices^9^. In contrast, M2-like macrophages are characterized by effectors, including Arg1, Ym1, and fizz1, which associate with responses to anti-inflammatory reactions and tissue remodeling^10,11^. Sepsis, often accompanied by multi-organ dysfunction syndrome with high mortality and morbidity^12^, is mainly mediated by M1-polarized macrophages, and inhibiting M1-polarized macrophages exhibits significant effect in sepsis.

Autophagy is a cellular degradation and recycling process, involving in various physiological processes including metabolism, survival, and host defense^13^. Recent studies demonstrate that autophagy is tightly contacted with immunity and inflammation^14^. Autophagy is a basic form of eukaryotes to protect from microbe invasion, and its absence leads to several pathological conditions, such as cancer and autoimmune disease^15^. Anti-inflammatory effects of autophagy display in macrophage proliferation, monocyte recruitment, and macrophage activation^16^, while the underly mechanism is still not clear. In septic mice, rapamycin treatment improved inflammatory status and rescued animals from septic death^17^, which indicated that autophagy activation maybe an essential strategy to regulate macrophage-induced inflammation in vivo. The potential mechanism and the prospective candidate for anti-inflammation need to be further studied.

Ubiquitin-specific proteases 14 (USP14) acts as proteasome-associated deubiquitinase, releasing ubiquitin from the proteasome and targeting ubiquitinated proteins, which is important in autophagy and inflammation. Deubiquitinating enzymes (DUBs) activity of USP14 was necessary for inflammasome activation, and inhibiting its activity blocked the release of IL-1β and caspase-1 activation^18^. In microbial infection, USP14 induced IκB degradation and promoted lipopolysaccharide (LPS)-mediated activation of NF-κB^19^. The known USP14 inhibitor, IU1, attenuated intrapulmonary inflammatory response and alleviate ventilator-induced rat lung injury^20^. These researches provide a potential anti-inflammatory target, USP14, but the exact mechanism of USP14 in autophagy and inflammation needs further studies.

S5, (Z)-1,3-dihydroxy-9-methyl-13 H-benzo [b] chromeno [3,2-f] [1,4] oxazepin-13-one F, is a chromone derivative. In our previous study, S5 improved contact hypersensitivity through activation of T lymphocytes^21^. In this paper, we first demonstrated that S5 greatly inhibited macrophage-induced inflammation directly via targeting USP14, resulting in ubiquitination of Beclin1 and cellular autophagy. Our study provides a new candidate compound, as autophagic modulator, for alleviating macrophage inflammation.

## Materials and methods

### Mice

Female C57BL/6 mice (6–8 weeks old, 18–22 g) were obtained from Model Animal Genetic Research Center of Nanjing University (Nanjing, China). They were maintained with free access to pellet food and water in plastic cages 21 ± 2 °C and kept on a 12 h light/dark cycle in specific pathogen-free facilities. Animal welfare and experimental procedures were carried out strictly in accordance with Guide for the Care and Use of Laboratory Animals (National Institutes of Health, the United States) and the related ethical regulations of Laboratory Animal Ethics Committee of School of Life Sciences, Nanjing University. Animal studies were in compliance with the ARRIVE (Animal Research: Reporting of In Vivo Experiments) guidelines. And all efforts were made to decrease animals’ suffering and to reduce the number of animals used in this manuscript.

### Regents

**Fig. 1:**
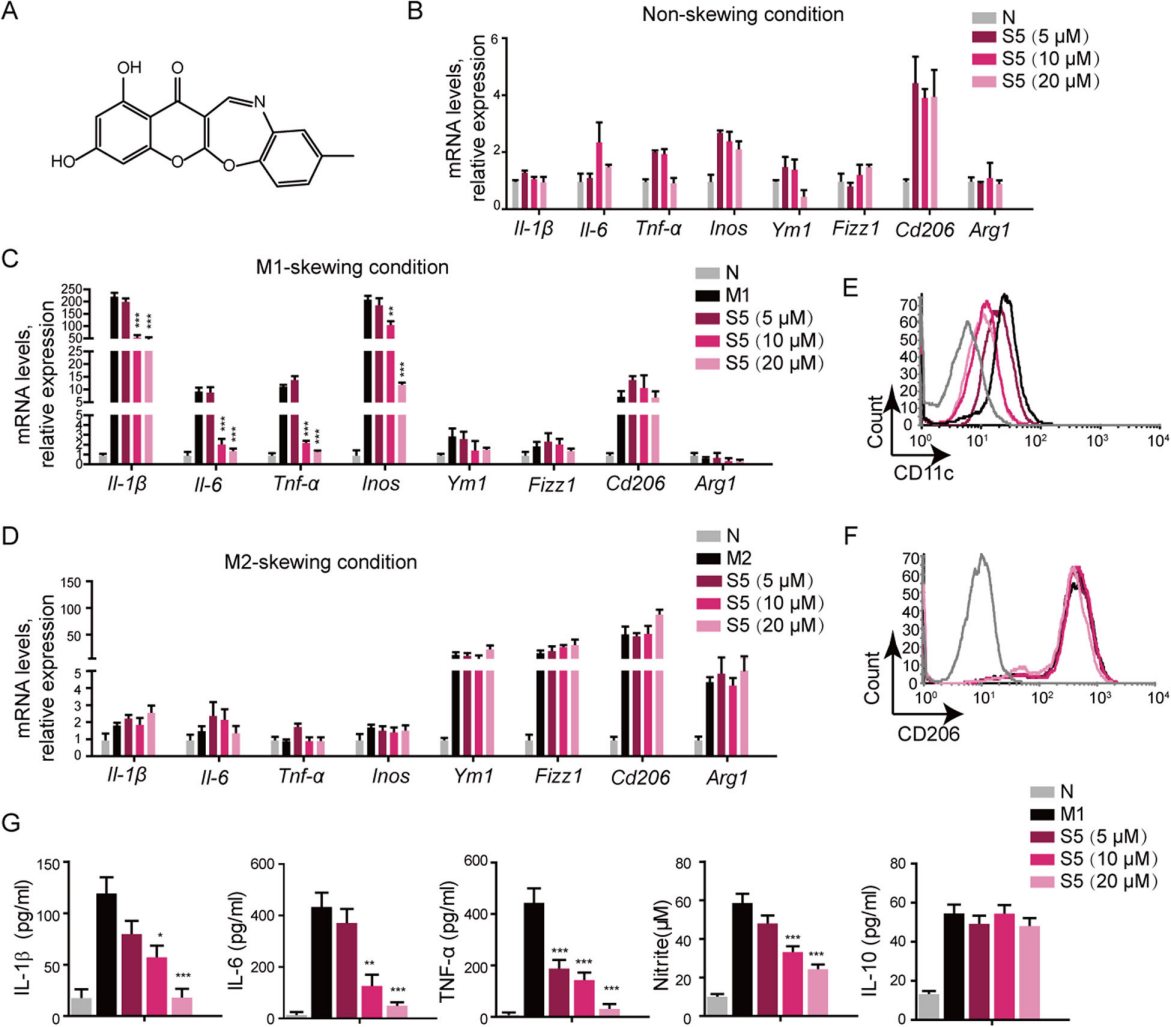
S5 inhibited macrophage M1-like polarization. **a** Chemical structure of neochromine S5. **b**–**d** mRNA levels of *Il-1β*, *Il-6*, *Tnf-α*, *Inos*, *Ym1*, *Fizz1*, *Cd206*, and *Arg1* in nonactivated BMDMs (**b**), M1-BMDMs (**c**) activated by 10 ng/mL LPS and 10 ng/mL IFN-γ for 6 h, and M2-BMDMs (**d**) activated by 20 ng/mL IL-4. Cells were treated with 5, 10, and 20 μM of S5 or the same volume of DMSO. Data are means ± SEM of five independent experiments. **P < 0.01, ***P < 0.001 vs. M1 group. **e**, **f** Flow cytometry results indicated that CD11c was decreased in M1-BMDMs and CD206 was unchanged in M2-BMDMs. **g** Cytokines level of IL-1β, IL-6, TNF-α, and IL-10 secreted in M1-BMDMs were assessed by ELISA, and nitrite was assessed by Griess regent. Data are means ± SEM of five independent experiments. *P < 0.05, **P < 0.01, ***P < 0.001 vs. M1 group.

Chemical structure of S5 ((Z)-1,3-dihydroxy-9-methyl-13 H-benzo [b] chromeno [3,2-f] [1,4] oxazepin-13-one F) was shown in Fig. 1a, and the synthetic method was performed as previously described^10^. S5 was dissolved at 100 mM in 100% DMSO as a stock solution, stored at −20 °C, and diluted with medium before every experiment. LPS were purchased from Sigma-Aldrich (St. Louis, MO). Recombinant human ubiquitin 7-amido-4-methylcoumarin (Ub-AMC, ab206162, Abcam). Recombinant human USP14 (Cat # E-544, R&D) and human 26 S proteasome (Ptsm, Cat # E-365, R&D). were purchased from R&D Systems Company. Annexin V-FITC/propidium iodide (AV/PI) assay kit for flow cytometry was purchased from Jingmei Biotech Co., Ltd. (Shenzhen, China). Recombinant murine IFN-γ (Cat # 315-05), IL-4 (Cat # 214-14), and macrophage colony-stimulating factor (M-CSF; Cat # 315-02) were purchased from Peprotech (Rocky Hill, NJ). 40,6-Diamidino-2-phenylindole (DAPI) were purchased from Invitrogen (Carlsbad, CA). ELISA kit for murine IL-1β, IL-6, and TNF-α were purchased from Dakewe Biotech Co. Ltd (Shenzhen, China). Clodronate liposome and PBS liposome were purchased from clodronateliposomes.com. Immunohistochemistry detection kit (Cat # GK500705) was purchased from GeneTech (Shanghai, China). Cell Counting Kit-8 (CCK-8; Cat # C0038) was purchased from Beyotime Biotechnology (Shanghai, China). All other chemicals were purchased from Sigma Chemical Co. (St. Louis, MO). Antibodies of western blot, flow cytometry, and co-immunoprecipitation (Co-IP) used in this study were described in Supplementary Table 2.

### Cell culture

Murine macrophage cell line RAW264.7 cells were purchased from the American Type Culture Collection (Rockville, MD), and maintained in DMEM (GIBCO, Grand Island, NY) containing 10% fetal bovine serum (GIBCO, Grand Island, NY), 100 U/mL penicillin, and 100 mg/mL streptomycin in 5% CO_2_ at 37 °C. They were authenticated and tested without mycoplasma contamination.

Bone marrow-derived macrophages (BMDMs) were obtained according to previously described^22^. In brief, using a 21-ga needle flushed femurs with PBS. Cells were grown in RPMI 1640 (GIBCO, Grand Island, NY) medium containing 10% fetal bovine serum and 20 ng/mL M-CSF in 5% CO_2_ at 37 °C for 5 days. Differentiated macrophages were washed by PBS two times and cultured with fresh DMEM medium containing 10% fetal bovine serum.

For M1 macrophage polarization, RAW264.7 cells and BMDMs were treated with 10 ng/mL LPS and 10 ng/mL IFN-γ for 6 h. For M2 macrophage polarization, RAW264.7 cells and BMDMs were treated with 10 ng/mL IL-4 for 6 h.

### MTT assay

Cells were seeded in 96-well plate and incubated with various concentrations of S5 in the presence or absence of 10 ng/mL LPS and 10 ng/mL IFN-γ for 24 h. Twenty microliters of MTT (4 mg/mL) dissolved in PBS was added to the wells in the plate and cultured at 37 °C for 4 h. The plate was centrifuged at 1200 × *g* for 5 min and the supernatant was discarded, 200 μL DMSO was added and then vibrated for 10 min. The absorbance was detected at 570 nm. Thereafter, 20 μL CCK-8 was added to the wells in the plate and then cultured at 37 °C for 2 h. The absorbance was detected at 450 nm.

### Cytokine analysis by ELISA

Mice blood sample was incubated at 37 °C for 30 min, and then centrifuged at 4500 r.p.m. for 15 min at 4 °C. Serum cytokines of IL-1β, IL-6, and TNF-α were measured using ELISA kits purchased from Dakewe Biotech Co. Ltd (Shenzhen, China) according to the manufacturer’s instructions.

### Quantitative PCR

Total RNA was extracted from cells or tissues by Trizol, and 1 μg mRNA was reverse transcribed to cDNA and subjected to quantitative PCR, which was performed with the BioRad CFX96 ouch^TM^ Real-Time PCR Detection System (Biorad, CA) using iQ^TM^ SYBR Green supermix (BioRad, CA), and threshold cycle numbers were obtained using BioRad CFX manager software. The program for amplification was 1 cycle of 95 °C for 2 min followed by 40 cycles of 95 °C for 10 s, 60 °C for 30 s, and 72 °C for 30 s. The level of β-actin was used to normalized the data. The primer sequences used in this study were described in Supplementary Table 1. The relative expression of each gene was normalized to the expression of β-actin, and then reported as fold change of basal level.

### Flow cytometry

Cultured cells were harvested or collected from BAFL, stained with specific antibodies for 30 min at 4 °C in dark, washed three times with cold PBS, and analyzed by flow cytometry.

### Western blot analysis

Western blot was performed according to described previously^23^. Cells were collected and lysed using lysis buffer, and quantitated by BCA assay. Then, obtained protein lysates were degenerated at 100 °C for 5 min, and separated by 10% SDS–PAGE and electrophoretically transferred onto polyvinylidene fluoride membranes (Millipore Corp, Bedford, MA). The membranes were blocked with 3% BSA for 1 h at room temperature, then incubated with specific primary antibodies overnight at 4 °C, and finally incubated with HRP-coupled secondary antibody. Protein bands were visualized using western bolting detection system according to the manufacturer’s instructions (Cell Signaling Technology, MA).

### Immunofluorescent microscopy

RAW264.7 cells were seeded on coverslips, and pretreated with S5 or the same volume of DMSO in the presence of LPS and IFN-γ. Six hours later, cells were fixed in 4% paraformaldehyde for 10 min at 37 °C, permeabilized with 0.5% Triton X-100 for 30 min, and blocked with 3% BSA for 1 h at room temperature, and stained with P65 and STAT1 antibody overnight at 4 °C, and then with specific fluorescent-coupled secondary antibody for 2 h. The coverslips were stained with DAPI and imaged with a confocal laser scanning microscope (Olympus Lake Success, NY). The quantification of CD11c, Ly6c, and Ly6g-positive cells in lung tissue were detected by immunofluorescence. Paraffin-embedded lung tissue were heat-fixed, deparaffinized, rehydrated, antigen retrieval before permeabilized, and subsequent process followed cells. Pictures were taken by single-blind method that the observer was unknown about the experimental design and grouping.

### Plasmids and siRNA transient transfection in cells

Plasmids and siRNA were transiently transfected to RAW264.7 cells using Lipofectamine 3000 (Invitrogen, Carlsbad, CA) according to the manufactures’ recommendation, then incubated at 37 °C for 48 h. In the end, the cells were collected and prepared for the following experiments.

The following plasmids used in this article were manufactured by Genscript Biotechnology: Flag-tagged Beclin1, HA-tagged Bcl2, His-tagged USP14, EGFP-USP14, EGFP-USP14-mut (S404A, F405A, and C414A), HA-TRAF6, and then were cloned into HindIII/EcoRI sites of pcDNA 3.1(+).

siRNA for mouse were purchased from Genepharma company.

siUSP14: 5′-AUAUAGUUGAAUCCAAGGAUAACCC-3′;

siBeclin1: 5′-GGAGCCAUUUAUUGAAACUTT-3′.

### Co-immunoprecipitation

RAW264.7 cells were incubated with various concentration of S5 at 100 mm dish for 6 h. The control group added same volume of DMSO. Harvested cell lysates were incubated with appropriate antibody at 4 °C overnight, and precipitated with protein A/G-agarose beads for another 4 h at 4 °C. The beads were washed by lysis buffer five times and centrifugated at 1000 × *g* for 5 min at 4 °C, and then immunoprecipitated proteins were detected by western blot.

### Cellular thermal shift assay

Cellular thermal shift assay (CETSA) was performed as described previously reported^24^. Briefly, RAW264.7 cells were incubated with 20 μM S5 or same volume DMSO for 6 h. Then cultured cells were harvested, and equally divided and lysed. Heating with various temperature via PCR instrument and then repeating freeze-thawing with liquid nitrogen three times, and centrifuging at 20,000 × *g* for 20 min at 4 °C. The supernatants were transferred to new microtubes and analyzed via western blot.

### Microscale thermophoresis

To determine solution equilibrium interaction constants between S5 and USP14, we used the microscale thermophoresis (MST) technology. S5 as ligand, and expressed EGFP-USP14 or EGFP-USP14 mutant (S404A, F405A, and C414A) in HEK293T cells as target, we diluted S5 to 16 different concentrations ranging from 30.5 ρM to 1 μM, and then mixed both equivalently at room temperature for 20 min. The mixture samples were loaded into capillaries (Monolith NT.115 Capillary) and the thermophoresis signals were measured using Nanotemper Monolith NT.115 (NanoTemper) according to the standard protocol of the manufacturer. The changes of the fluorescent thermophoresis and *K*_d_ values were analyzed using the Nano Temper analysis software.

### Ub-AMC hydrolysis assay

Ub-AMC was purchased from Boston Biochem. The deubiquitination assay was performed by previous published protocol^25^. Briefly, the reaction system contained 50 mM Tris-HCl (pH 7.5), 1 mM EDTA, 1 mM ATP, 5 mM MgCl_2_,1 mM DTT, 1 mg/mL ovalbumin, 1 nM proteasome, and 40 nM USP14, various concentrations of S5 were added to the system in a 50 μL reaction volume. The reaction was started by adding 1 μM Ub-AMC to the system and measured at Ex345/Em445 using an Envision plate reader (PerkinElmer). Fluorescence intensity was recorded every 30 s for 20 min. Each experiment was repeated three times, and the average value was calculated.

### CLP-induced sepsis

Mice were randomly assigned to five experimental groups (*n* = 10 mice per group) using the random array after acclimating to the housing environment for 7 days: (1) Sham, (2) vehicle with cecal ligation and puncture (CLP) mice (CLP), (3) S5 at 5 mg/kg with CLP mice, (4) S5 at 10 mg/kg with CLP mice, and (5) S5 at 20 mg/kg with CLP mice. Data collection and analysis were performed blindly, the experimenters were unaware of the group assignment and animal treatment.

CLP surgery was performed as described previously^26^. Mice were administered with S5 (5, 10, and 20 mg/kg) orally before suffering CLP surgery. Survival rate was observed and calculated. The animals were killed by overdose sodium pentobarbital (300 μL/20 g body weight 2% sodium pentobarbital intraperitoneally injection), serum, bronchoalveolar lavage fluid (BALF), and lung tissue were collected 6 h after CLP challenge. Lung sample was fixed with formaldehyde and microscopically examined for inflammatory infiltration by hematoxylin and eosin staining with single-blind method that the observer did not know the experimental design and grouping.

### Macrophages depletion and pretreated BMDMs adoptive transfer

Systemic macrophages were depleted using clodronate liposome by intraperitoneal injection in C57BL/6 mice, 100 μL per 20 g body weight. After 24 h, the proportion of CD11b-positive macrophage populations in peritoneal cells was measured by flow cytometry to determine the depletion efficiency. BMDMs were polarized to proinflammatory M1 phenotype by treating it with 10 ng/mL LPS and 10 ng/mL IFN-γ or 20 μM S5 or same volume of DMSO for 6 h, and normal BMDMs were also treated with the same volume of DMSO. Pretreated cells were transferred into macrophage-depleted mice (1 × 10^6^ cells 100 μL per mice and per 20 g body weight, i.v.). Two hours later, C57BL/6 mice were administered (i.p.) 10 mg/kg LPS and the survival rate was monitored at different times. Survival rate was recorded for 60 h until the mice died. Under anesthesia, blood samples were collected 4 h after LPS challenge.

### BALF collection and detection

After suffered sepsis, mice were subjected to BAL using 0.5 mL PBS for three times. Then BALF was centrifuged at 250 × *g* for 5 min at 4 °C (ref. ^27^). Using commercial ELISA kits detected cytokines of the supernatants of BALF, and western blots detected proteins expression of cells from BALF.

### Statistical analysis

All experiments are randomized and statistical analysis was performed with GraphPad Prism 5.0 software (San Diego, CA, USA). All data were presented as mean ± SEM of the mean from three independent experiments with each experiment including triplicate sets. Results were presented as means ± SEM, and *n* = 10 mice for every experimental group in vivo. Grubbs test was used to exclude samples from analysis. One-way ANOVA analysis and Student’s *t*-test were used for comparison among various experimental and control groups. Student’s *t*-test was used to compare between two groups. *P*-value < 0.05 was considered to be statistically significant.

## Results

### S5 inhibits M1-like macrophage polarization, without influence in M2-like polarization

The chemical structure of S5 was shown in Fig. 1a. MTT assay and AV/PI staining demonstrated that S5 had no obvious toxic effect in either resting or LPS- and IFN-γ-stimulated BMDMs (Supplementary Fig. S1a, b). S5 dose-dependently inhibited M1-like macrophages-related mRNA levels, but with no effect on resting or M2-like macrophages. (Fig. 1b–d). Besides, the proportion of CD11c (surface marker of M1-like macrophages)-positive cells was obviously decreased by S5 treatment (Fig. 1e), while CD206 (surface marker of M2-like macrophages)-positive cells were not affected (Fig. 1f). ELISA assay showed that S5 significantly restrained IL-1β, IL-6, TNF-α, and nitrite protein levels secreted by BMDMs (Fig. 1g) and RAW264.7 cells (Supplementary Fig. S1c). These results suggest that S5 greatly inhibits macrophage M1-like macrophages polarization with no effect on M2-like macrophages in vitro. We further detected the classic signaling pathway of M1 polarization, including STAT1, NF-κB, and MAPK. In the stimulation of LPS and IFN-γ, S5 significantly and dose-dependently inhibited the phosphorylation of NF-κB P65, STAT1, and JAK1 (Supplementary Fig. S2a, b). Consistently, immunofluorescence analysis demonstrated that S5 obviously suppressed nucleus translocation of P65 (Supplementary Fig. S2e, f) and STAT1 (Supplementary Fig. S2g, h) at 20 μM, suggesting that S5 downregulated STAT1 and P65 activity, which was made sense with the data of western blot. However, the effect on MAPK signaling pathway was too weak, and phosphorylated p38, ERK, and JNK did not change by S5 treatment (Supplementary Fig. S2c, d). These results suggest that S5 mainly inhibits NF-κB and STAT1 signals in the classic activation of M1-like macrophages.

### S5 protects mice from CLP-induced sepsis through typically inhibiting M1-like macrophage polarization

**Fig. 2:**
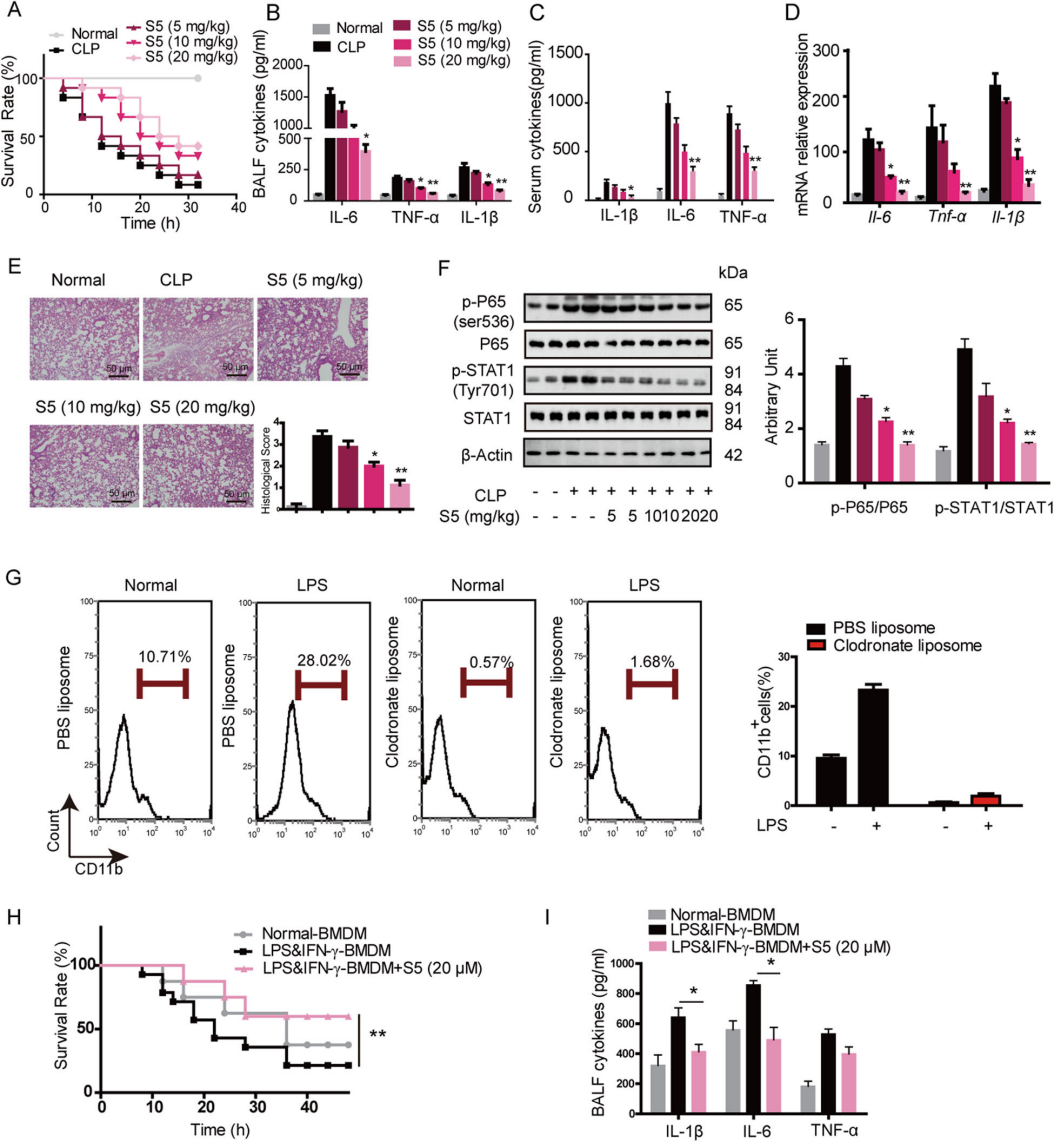
S5 protected mice from sepsis via inhibition of M1 polarization. **a** Various dose of S5 or vehicle (CMC-Na) intragastric administrated 2 h before suffering CLP surgery in C57BL/6 mice. Survival rate was observed and calculated (*n* = 10 mice per group). Data are means ± SEM, *n* = 10 mice for each experimental group. **P < 0.01 vs. CLP group. **b**–**d** Mice were treated as **a**. Four hours later, cytokines of BALF (**b**) and serum (**c**) were determined by ELISA, mRNA level of cytokines in lung tissue was determined by RT-PCR (**d**). Data are means ± SEM, *n* = 10 mice for each experimental group. *P < 0.05, **P < 0.01 vs. CLP group. **e** Hematoxylin and eosin staining of lung sections, scale bar 50 μm. Data are means ± SEM, *n* = 10 mice for each experimental group. *P < 0.05, **P < 0.01 vs. CLP group. **f** p-P65, p65, p-STAT1, STAT1, and β-actin expression in cells of BALF were detected by western blot. Data are means ± SEM, *n* = 10 mice for each experimental group. Two samples of each group were shown as representatives. *P < 0.05, **P < 0.01 vs. CLP group. **g** Clodronate (100 μL per 20 g body weight) or PBS liposome was i.p. administered, 24 h later, mice were challenged by LPS (10 mg/kg) for 6 h and the expression of CD11b in peritoneal exudate cells was detected by flow cytometry. Data summary were expressed as a histogram of mean ± SEM, *n* = 6 mice for each experimental group. **h** Clodronate (100 μL per 20 g body weight) liposome was i.p. administered in mice 24 h before. BMDMs were incubated with S5 at 20 μM or the same volume of DMSO in the presence of 10 ng/mL LPS and 10 ng/mL IFN-γ for 6 h in vitro. The pretreated cells were harvested and washed three times with cold PBS. Macrophage-depleted mice were treated with normal BMDMs, LPS and IFN-γ-BMDMs, or LPS and IFN-γ-BMDMs with S5 treatment (1 × 10^6^ cells per 20 g, 100 μL per mice, i.v.) 2 h before LPS (10 mg/kg, i.p.) challenged (i.v.). Survival rate was observed and calculated for 48 h until there were no more deaths. Data are means ± SEM, *n* = 10 mice for each experimental group. **P < 0.01 vs. LPS and IFN-γ-BMDM group. **i** Mice were administered as **h**, 4 h after LPS challenge, BALF cytokines were measured by ELISA. Data are means ± SEM, *n* = 10 mice for each experimental group. *P < 0.05 vs. LPS and IFN-γ-BMDM group.

In the progression of sepsis, M1-like macrophages play a significant role and generate a series of inflammatory response. Our studies found that in CLP-induced murine sepsis, simultaneous administration with S5 significantly increased the survival rate of mice from 5 to 45% (Fig. 2a). S5 also dramatically suppressed the levels of proinflammatory cytokines IL-1β, IL-6, and TNF-α in serum, and BALF and mRNA levels in lung tissue (Fig. 2b–d). Histopathological analysis showed S5 improved the lung lesion of septic mice with descent of tissue damage and reduced inflammatory cells infiltration (Fig. 2e). Western blot revealed that S5 obviously inhibited phosphorylation of P65 and STAT1 (Fig. 2f).

To further confirm S5 relieved CLP-induced sepsis via inhibiting M1-like macrophages polarization, we conducted macrophage depletion in mice by clodronate liposome, and then the mice were challenged with CLP-induced sepsis. The depletion efficiency was examined by staining with a macrophage surface marker CD11b (Fig. 2g). Then, the pretreated macrophages were adoptively transferred into macrophage-depleted mice as shown in Fig. 2h. Results indicated that the mice transferred with S5-pretreated M1-BMDMs showed significantly higher survival rate than the mice transferred with M1-BMDM alone. We also detected levels of proinflammatory cytokines (IL-1β, IL-6, and TNF-α) in BALF. The data showed that proinflammatory cytokines in mice transferred with S5-pretreated M1-BMDMs was dramatically reduced than in the control group (Fig. 2i). These results display that S5 improves CLP-induced sepsis through inhibiting M1 polarization.

**Fig. 3:**
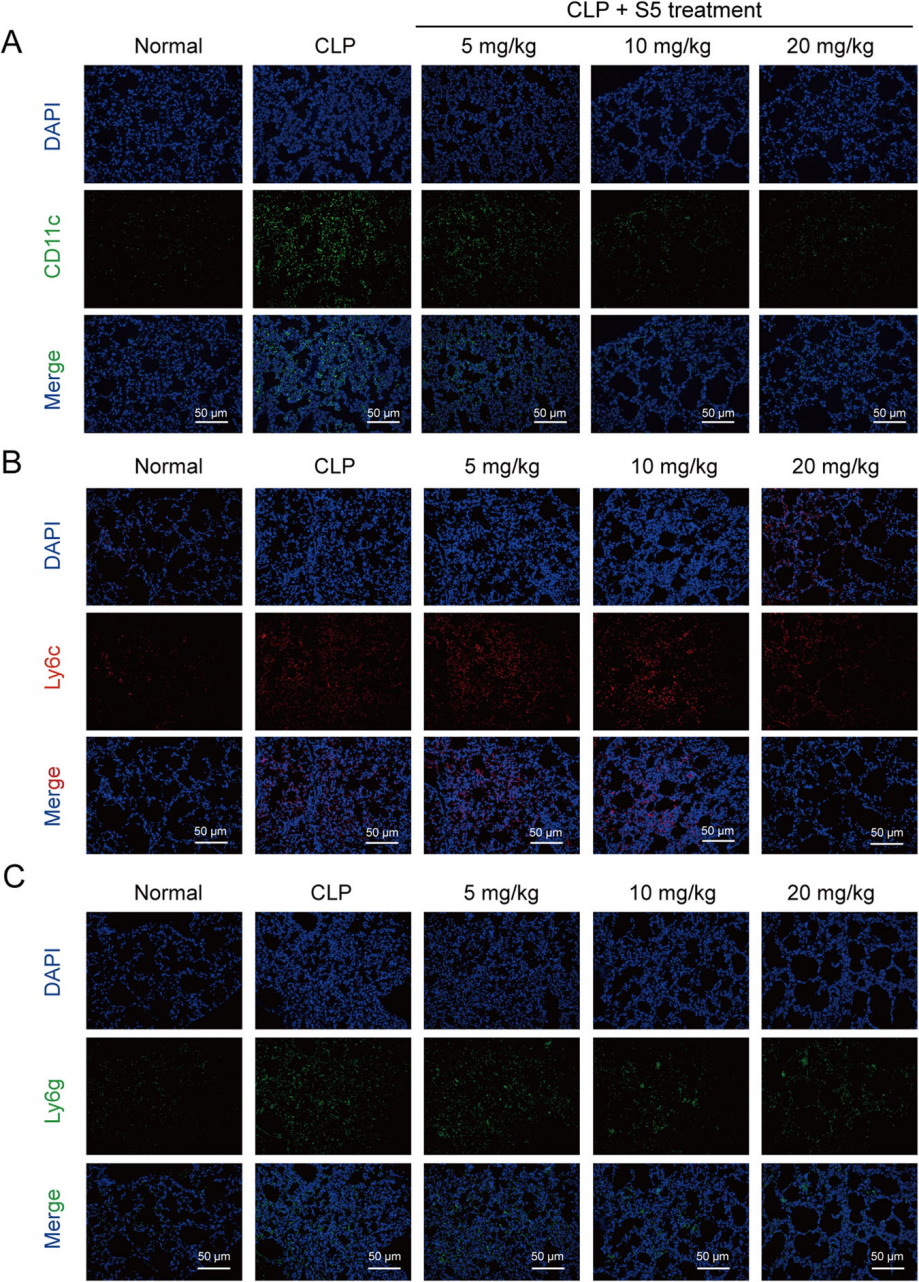
S5 reduced the M1-like macrophages infiltration in septic lung tissue. Mice were treated as in Fig. 2. Lung tissues were collected after mice suffering CLP surgery. **a** CD11c, **b** Ly6c, and **c** Ly6g expression in lung tissue were detected by immunofluorescence. Scale bar 50 μm. The shown picture was a representation of three images within random fields of every sample.

Furthermore, immunofluorescence showed that S5 significantly reduced the percentage of CD11c^+^ cells in lung tissue after CLP surgery, while with slight effects in Ly6g^+^ (neutrophils) and Ly6c^+^ (monocytes) cells (Fig. 3a–c).

### S5 induces cellular autophagy that is responsible for the inhibition of M1-like macrophages polarization

**Fig. 4:**
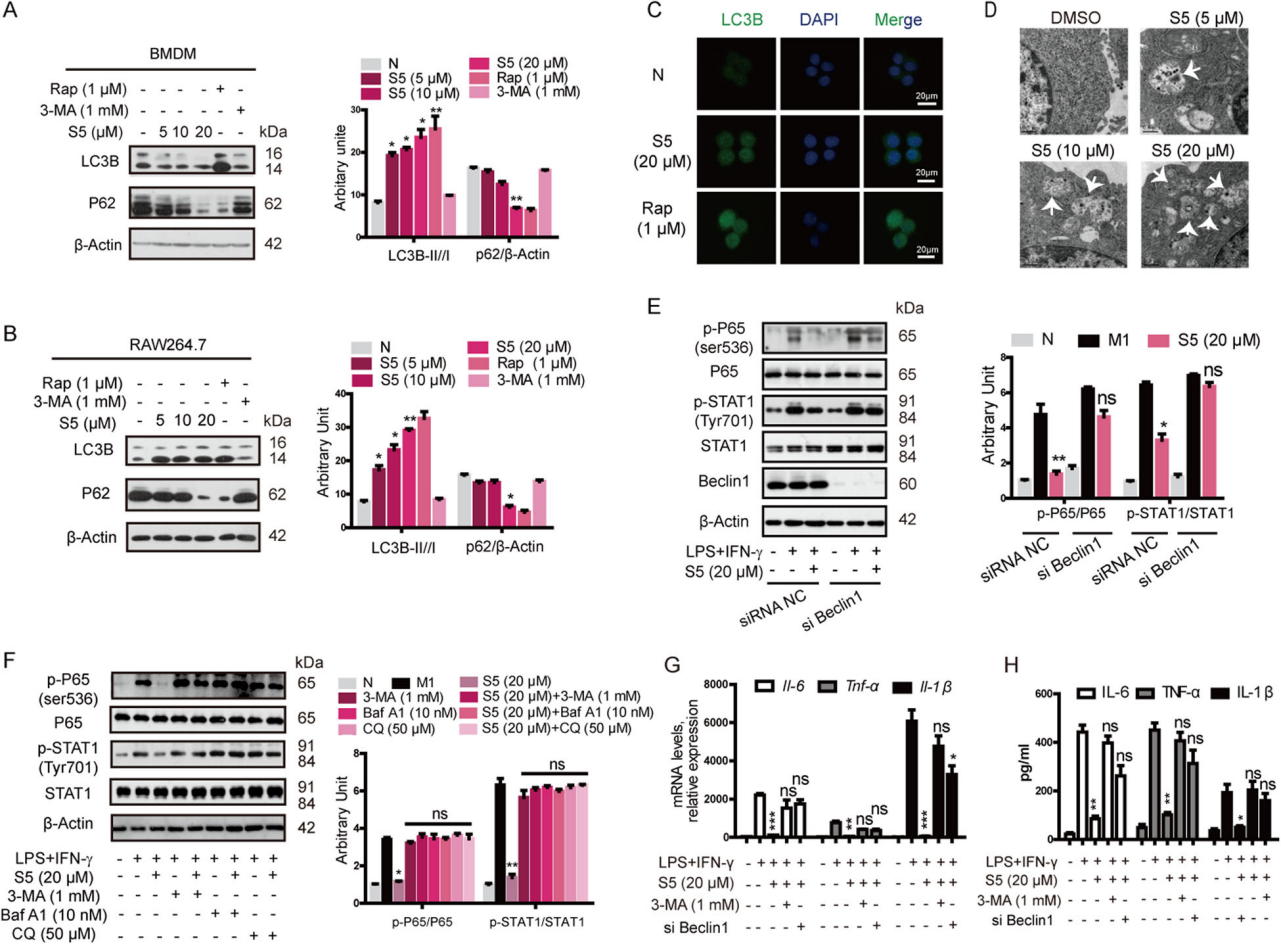
S5 increased macrophage autophagy in vitro. **a**, **b** BMDMs and RAW264.7 cells were treated with 5, 10, and 20 μM of S5 and 1 μM of rapamycin, 1 mM 3-MA or the same volume of DMSO, respectively, for 6 h. S5 promoted autophagy by enhancing LC3B-II/I ratio and decreasing P62 expression. Data are means ± SEM of five independent experiments. *P < 0.05, **P < 0.01 vs. DMSO group. **c** LC3B expression in RAW264.7 cells were analyzed by immunofluorescence staining. Scale bar, 20 μm. **d** BMDMs were fixed and examined for autophagosome formation by transmission electron microscopy. Scale bar, 0.5 μm. **e**, **f** The inhibition effect of S5 on M1 polarization signal pathway was repressed with Beclin1 knockdown (**e**) or autophagic inhibitor 3-MA, BafA1, and CQ (**f**). Data are means ± SEM of five independent experiments. ns nonsignificant, *P < 0.05, **P < 0.01 vs. LPS and IFN-γ group. **g**, **h** Il-6, TNF-α, and Il-1β mRNA expression were detected by RT-PCR (**g**), and protein level in medium was detected by ELISA assay (**h**). Data are means ± SEM of five independent experiments. ns nonsignificant, *P < 0.05, **P < 0.01, ***P < 0.001 vs. LPS and IFN-γ group.

To assess the effect of S5 on autophagy, we detected the autophagic protein expression, including LC3B-II//I ratio, Beclin1, and P62. S5 dramatically upregulated LC3B-II/I ratio and Beclin1 expression, and downregulated P62 expression (Fig. 4a, b). Immunofluorescence of LC3B (Fig. 4c) and electron microscope analysis (Fig. 4d) indicated that S5 significantly stimulated the formation of autophagosomes in RAW264.7 cells, as shown in white arrows.

Beclin1 was considered as a key activator of early autophagosome assembly^28^. RAW264.7 cells were transfected with a Beclin1 siRNA or control siRNA, and the interference efficiency was shown in Fig. 4e. Results indicated that the suppression of S5 on P65 and STAT1 phosphorylation in M1-like macrophages was reversed in RAW264.7 cells transfected with Beclin1 siRNA (Fig. 4e) or treated with autophagic inhibitors, 3-MA, BafA1, or CQ (Fig. 4f). Likewise, downregulation of S5 in inflammatory cytokines was also blocked by Beclin1 siRNA and 3-MA (Fig. 4g, h).

**Fig. 5:**
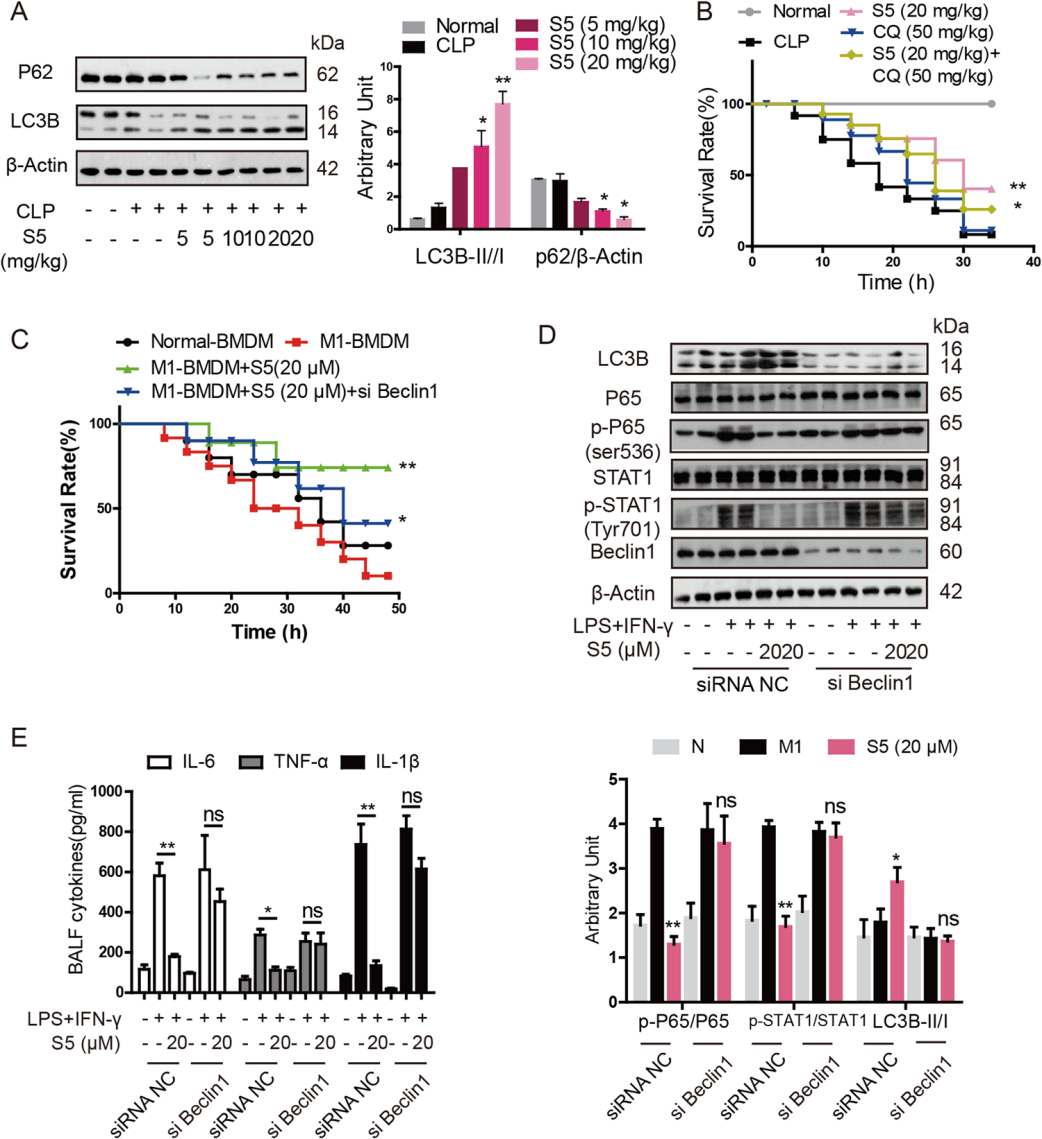
The improvement of S5 in CLP sepsis was greatly dependent on autophagy. **a** The same samples of Fig. 2f were used to detect p62 and LC3B expression by western blot. β-actin was used as loading control. Data are means ± SEM, *n* = 10 mice for each experimental group. Two samples of each group were shown as representatives. *P < 0.05, **P < 0.01 vs. CLP group. **b** Intragastric administrated C57BL/6 mice with 20 mg/kg S5 and 50 mg/kg CQ for 2 h before suffering CLP surgery. Survival rate was observed and calculated (*n* = 10 mice per group). Data are means ± SEM, *n* = 10 mice for each experimental group. *P < 0.05, **P < 0.01 vs. CLP group. **c** In vitro, BMDMs were incubated with 20 μM S5 or the same volume of DMSO in the presence of 10 ng/mL LPS with 10 ng/mL IFN-γ for 6 h, or previous knockdown of Beclin1 for 36 h. Macrophage-depleted mice were treated with normal-BMDM, LPS and IFN-γ-BMDM, or S5-treated LPS and IFN-γ-BMDM and S5-treated LPS and IFN-γ-BMDM with Beclin1 knockdown (i.v.), and challenged with 10 mg/kg LPS (i.p.). Survival rate was observed and calculated. Data are means ± SEM, *n* = 10 mice for each experimental group. *P < 0.05, **P < 0.01 vs. M1 + BMDM group. **d** P62, LC3B, p-P65, P65, p-STAT1, and STAT1 expression in cells of BAFL were detected by western blot. Data are means ± SEM, *n* = 10 mice for each experimental group. ns nonsignificant, *P < 0.05, **P < 0.01 vs. M1 + BMDM group. **e** Mice were treated as Fig. 4c, 4 h later, BAFL cytokines were measured by ELISA. Data are means ± SEM, *n* = 10 mice for each experimental group. ns nonsignificant, *P < 0.05, **P < 0.01 vs. M1 + BMDM group.

Furthermore, it was also confirmed S5 induced autophagy of macrophages in vivo. Results of western blot revealed that S5 increased autophagy in BALF macrophages of septic mice because of the dose-dependent upregulation of LC3 II/I and downregulation of P62 (Fig. 5a). And, CQ, the autophagy inhibitor, specially blocked the improvement of S5 in septic mice (Fig. 5b). We also performed adoptive transfer experiment and macrophages were pretreated as shown in Fig. 5c. Results indicated that the Beclin1 knockdown significantly restrained the improvement of S5 in septic mice and inflammatory responses (Fig. 5c–e). These results reveal that S5 promotes macrophage autophagy both in vitro and in vivo, which facilitates inhibition of M1 polarization and alleviation in inflammation.

### S5 subsequently increases Beclin1 ubiquitination and disturbs Beclin1–Bcl2 interaction, eventually leading to the upregulation of autophagy

**Fig. 6:**
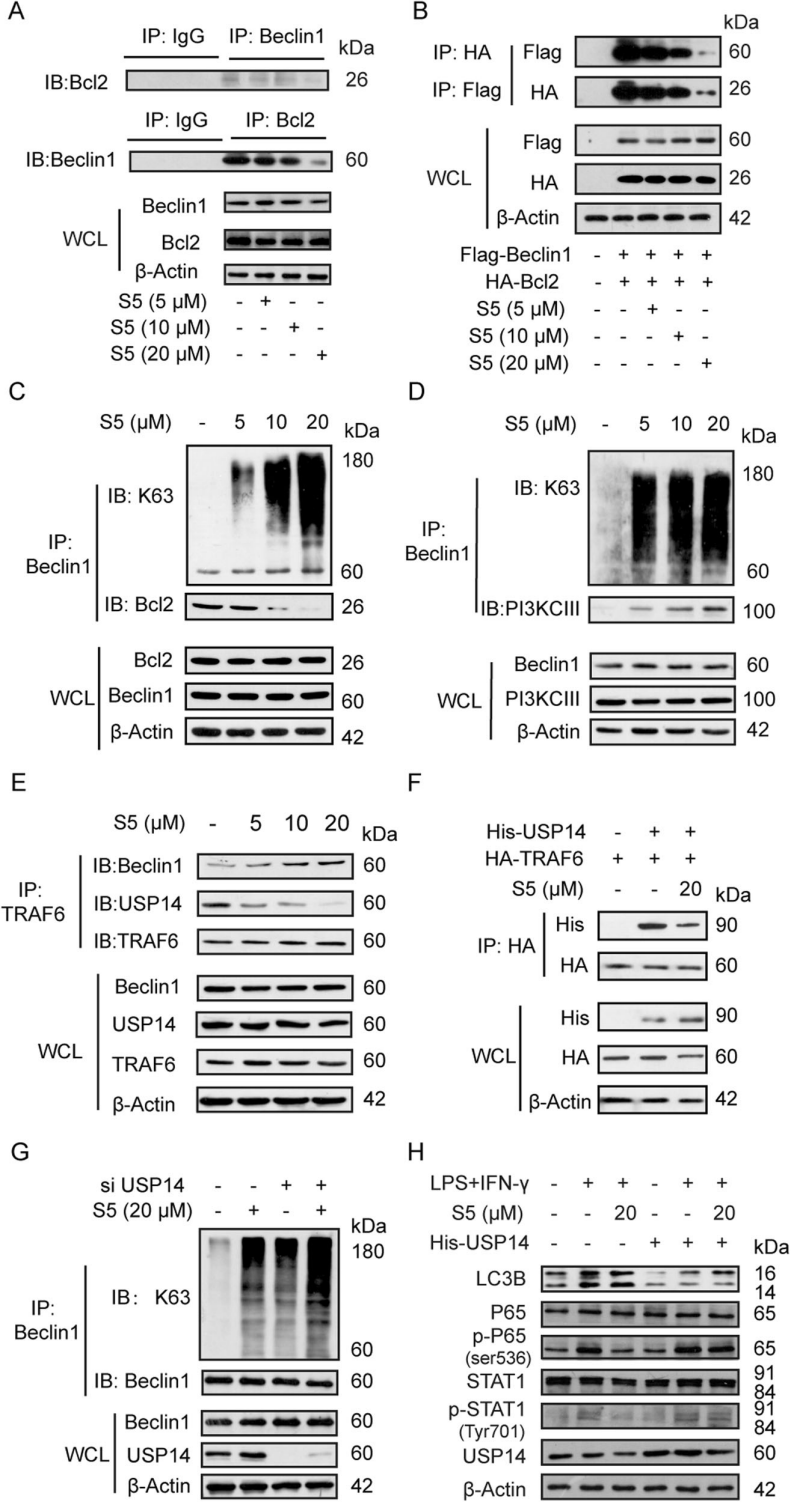
S5 increases Beclin1 ubiquitination and disturbs Beclin1–Bcl2 interaction. **a** RAW264.7 cells were incubated with various dosages of S5 (5, 10, and 20 μM) for 6 h. Co-immunoprecipitation was used to analyze Beclin1–Bcl2 interaction. **b** HEK293T cells were transfected with Flag-Beclin1 and HA-Bcl2 plasmids for 48 h, and incubated with various dosages of S5 for 6 h. Co-immunoprecipitation was used to analyze the interaction of Flag-Beclin1 and HA-Bcl2. **c**, **d** RAW264.7 cells were incubated with various dosages of S5 for 6 h. Co-immunoprecipitation experiments analyzed K63-ubiquitination of Beclin1 and the interaction of Beclin1 with bcl-2 or PI3KCIII. **e** RAW264.7 cells were incubated with various dosages of S5 for 6 h. Co-immunoprecipitation experiments analyzed the interaction of TRAF6 with USP14 or Beclin1. **f** HEK293T cells were transfected with HA-TRAF6 and His-USP14 for 48 h, and incubated with S5 (20 μM) for 6 h. Co-immunoprecipitation was used to analyze the interaction of HA-TRAF6 and His-USP14. **g** RAW264.7 cells were transfected with siRNA of USP14 or si-Control for 48 h, and co-immunoprecipitation was used to analyzed K63-ubiqutination of Beclin1 with or without S5 treatment. **h** RAW264.7 cells were transfected with His-USP14 plasmid for 48 h. P62, LC3B, p-P65, P65, p-STAT1, and STAT1 expression were detected by western blot. The relative statistics of **h** was shown in Supplementary Fig. S4. Data are means ± SEM of five independent experiments. ns nonsignificant, *P < 0.05 vs. LPS and IFN-γ group.

In following study, we focused on the main target of S5 in autophagy. Beclin1–Bcl2 interaction was the best negative regulator mechanism of the autophagy function^29,30^. Firstly, S5 was confirmed to dose-dependently interrupt Beclin1–Bcl2 interaction in Co-IP experiments, with both endogenous and overexpressed proteins (Fig. 6a, b). It was reported that K63-linked ubiquitination of Beclin1 disrupted Beclin1–Bcl2 interaction and drove autophagy during responses to inflammation^31^. Consequently, our results exhibited S5 treatment significantly promoted the K63-linked ubiquitination of Beclin1, reducing Beclin1–Bcl2 interaction (Fig. 6c) and enhancing Beclin1–class III PI3K interaction in RAW264.7 cells (Fig. 6d), simultaneously.

Furthermore, USP14, a DUB, was verified to induce deubiquitination of Beclin1 (ref. ^32^), while TRAF6, a ubiquitin-ligase, was responsible for the K63-linked polyubiquitination of Beclin1 (refs. ^33,34^). Our results showed that S5 dose-dependently decreased USP14–TRAF6 interaction, while increased TRAF6–Beclin1 interaction simultaneously (Fig. 6e). And the inhibitory effect of S5 on USP14–TRAF6 interaction was also confirmed in HEK293T cells transfected with His-USP14 and HA-TRAF6 (Fig. 6f).

In addition, the induction of S5 in Beclin1 ubiquitination and following autophagy was obviously blocked by USP14 knockdown, indicating that S5 exerted upregulation in autophagy mainly via USP14 (Fig. 6g and Supplementary Fig. S7). And USP14 knockdown in RAW264.7 cells reduced *Il-1β*, *Il-6*, and *Tnf-α* mRNA level, and depressed p-P65 and p-STAT1 expression compared to LPS and IFN-γ treatment, suggesting USP14 knockdown inhibited M1-like macrophage polarization (Supplementary Fig. S3a, b). Besides, USP14 overexpression also partially weakened the induced-autophagic and anti-inflammatory effect of S5 (Fig. 6h and Supplementary Fig. S4).

These results indicate that effect of S5 in Beclin1 polyubiquitination, Bcl2 and Beclin1 dissociation, autophagy activation, and M1 polarization inhibition is significantly dependent on USP14.

### S5 directly targets USP14 at Ser404, Phe405, and Cys414 to inhibit its function

**Fig. 7:**
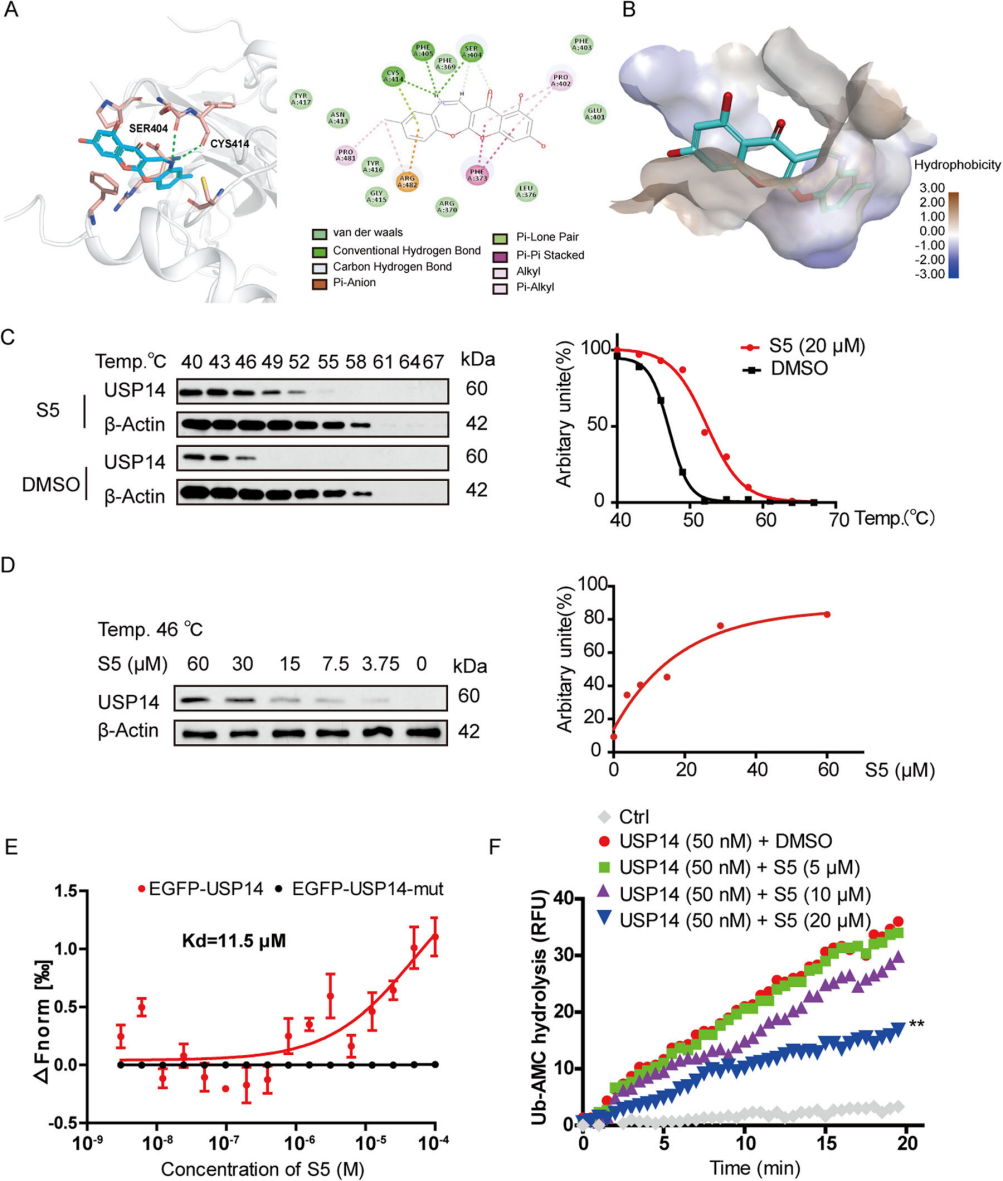
S5 directly targeted USP14 at Ser404, Phe405, and Cys414 to inhibit its function. **a** Molecular docking analysis of S5 and USP14 at different domains. **b** The shape and polarity of S5 binding pocket surface. **c**, **d** RAW264.7 cells were incubated with DMSO or S5 for 6 h, then the thermal stabilization of USP14 were analyzed by CETSA at different temperatures (**c**) and series of concentrations (**d**). **e** To further determine the interaction between S5 and USP14, MST experiment was carried out with a series concentration of S5 and EGFP-USP14 or EGFP-USP14 mutant (S404A, F405A, and C414A). Results showed that the *K*_d_ value of binding affinity for S5 and EGFP-USP14 was 11.5 μM. And the binding capacity between S5 and USP14 mutant (S404A, F405A, and C414A) was obviously blocked. **f** Ub-AMC hydrolysis assay was performed to evaluate effect of S5 on deubiquitinating enzymic activity of USP14 according to the instruction described in “Materials and methods” section. Fluorescence intensity was recorded every 30 s for 20 min. Each experiment was repeated three times, and the average value was calculated.

Then, we executed Discovery Studio and CETSA to validate the direct binding of S5 and USP14. Discovery Studio results predicted that S5 might form hydrogen binds with Ser404, Phe405, and Cys414 in the FERM domain of USP14 (PDB ID: 6IIK; Fig. 7a, b). CETSA revealed that S5 enhanced thermal stability of USP14 compared with DMSO treatment (Fig. 7c). And by keeping constant temperature at 46 °C, we found that USP14 protein level gradually enhanced in the concentration-dependent manner of S5 (Fig. 7d). To further determine the interaction between S5 and USP14, MST experiment was carried out. Results showed that the *K*_d_ value of binding affinity for S5 and EGFP-USP14 was 11.5 μM. And most importantly, the binding capacity between S5 and USP14 mutant (S404A, F405A, and C414A) was obviously blocked (Fig. 7e). Next, Ub-AMC hydrolysis assay was performed to evaluate the effect of S5 on deubiquitinating enzymic activity of USP14, and result showed that S5 at 20 μM inhibited activity of USP14, while 5 and 10 μM of S5 had no significant effect (Fig. 7f).

In addition, results in Supplementary Fig. S5 showed that expression of USP14 in M1 and M2 macrophages had no difference, and S5 did not affect USP14 protein level. Besides, USP14 mutant (S404A, F405A, and C414A) moderately suppressed the interaction with TRAF6, compared to USP14-WT (Supplementary Fig. S6).

### S5 exhibits higher efficiency and lower toxicity than IU1, the known USP14 inhibitor, which indicates that S5 may be the prospective candidate for anti-inflammation treatment via inducing autophagy

**Fig. 8:**
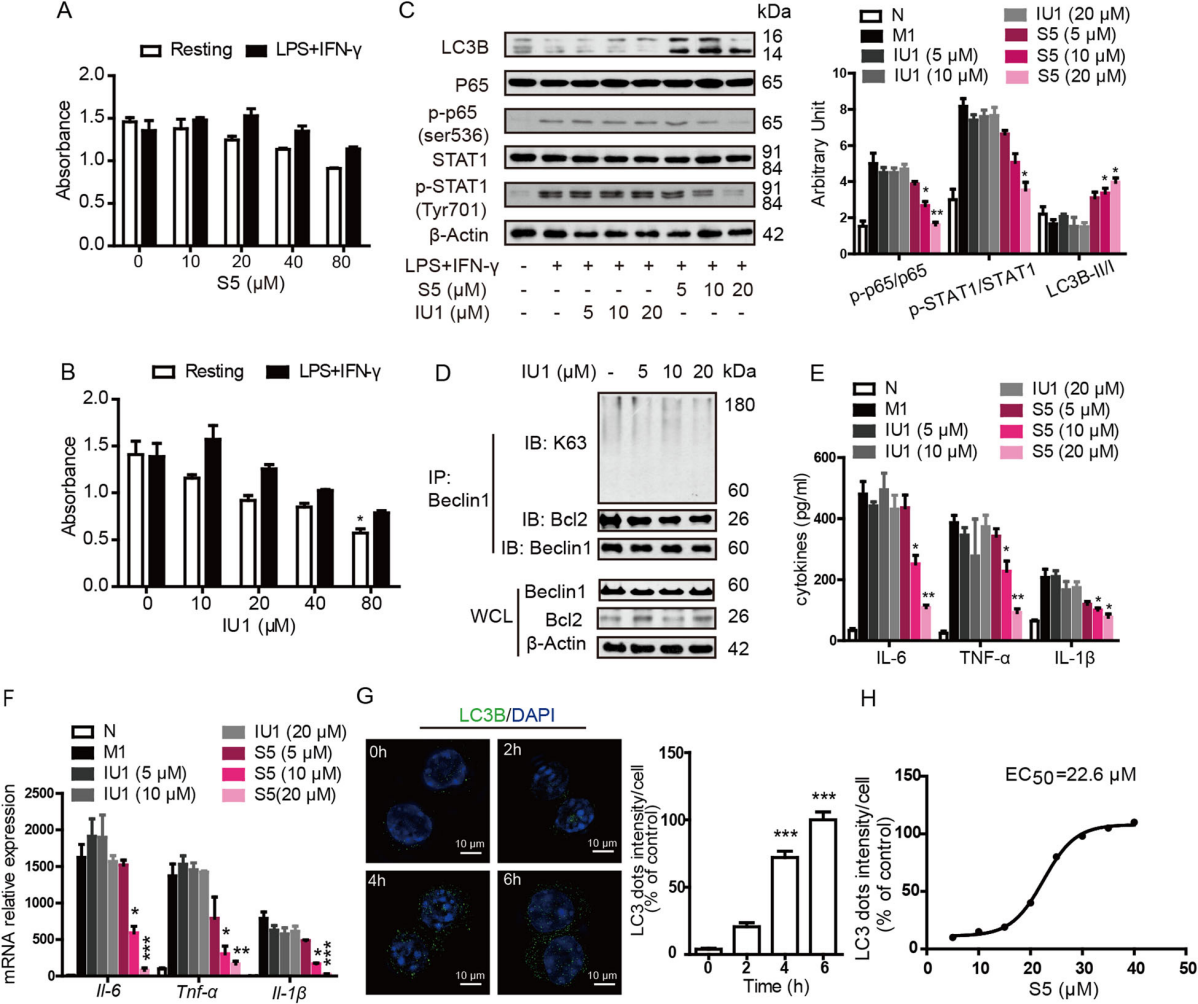
S5 was more efficient than IU1 in promoting autophagy. **a**, **b** RAW264.7 cells were incubated with various concentrations of S5, IU1, or the same volume of DMSO in the absence or presence of LPS and IFN-γ at 10 ng/mL for 24 h. Cytotoxicity was detected by CCK-8 assay. Data are means ± SEM of five independent experiments. *P < 0.05 vs. DMSO group. **c** RAW264.7 cells were treated as in Fig. 7a, P62, LC3B, p-P65, P65, p-STAT1, and STAT1 expression were detected by western blot. Data are means ± SEM of five independent experiments. *P < 0.05, **P < 0.01 vs. LPS and IFN-γ group. **d** RAW264.7 cells were incubated with 5, 10, and 20 μM of IU1, co-immunoprecipitation experiments showed the K63-linked ubiquitination of Beclin1. **e**, **f** RAW264.7 cells were treated as above, and IL-6, TNF-α, and IL-1β protein level in medium were detected by ELISA assay (**e**) and mRNA expression were detected by RT-PCR (**f**). Data are means ± SEM of five independent experiments. *P < 0.05, **P < 0.01, ***P < 0.001 vs. LPS and IFN-γ group. **g** RAW264.7 cells incubated with 20 μM of IU1 at 0, 2, 4, and 6 h, LC3B expression was analyzed by immunofluorescence staining. The average spot intensity in 1000 cells from each indicated sample was determined. Data are means ± SEM of five independent experiments. ***P < 0.001 vs. DMSO group. **h** RAW264.7 cells were incubated with various concentration of S5. The LC3 dots intensity was determined as in **g**.

We studied the differences between S5 and IU1, the known USP14 inhibitor, in regulating autophagy and inflammation. Results showed that the toxicity of IU1 was more severe than S5 at the same concentration both in resting and activated macrophages (Fig. 8a, b). In addition, we detected M1-associated signaling, and S5 appeared better anti-inflammatory effects than IU1(Fig. 8c, e, f). Besides, the ability of K63-linked ubiquitination induction was very weak at the same concentration of IU1 (Fig. 8d). IU1 was able to promote autophagy, but LC3 dots intensity achieved 100% till 24 h treatment, and the EC50 value of IU1 to induce autophagy was 63.4 μM (ref. ^32^). While, S5 was more efficiency than IU1, LC3 dots intensity achieved 100% at 6 h (Fig. 8g) and the EC50 value of S5 was 22.6 μM (Fig. 8h).

## Discussion

Sepsis is mainly characterized by M1-like macrophage activation. In this study, we provided new insights in the effect and mechanism of S5 in CLP-induced sepsis. In vitro, our results showed that S5 inhibited M1-like macrophages polarization, including STAT1/NF-κB signaling pathway and inflammatory cytokines release (Fig. 1 and Supplementary Fig. S1). In vivo, S5 alleviated CLP-induced sepsis, improving mortality, ameliorating lung inflammation, and maintaining intact lung tissue (Fig. 2a, e). Moreover, our results verified that S5 selectively targeted macrophages (Figs. 2h, i and 3a), not monocytes (Fig. 3b) or neutrophils (Fig. 3c) in sepsis.

Then, further studies indicated that S5 significantly induced the formation of autophagosomes, and Beclin1 knockdown or autophagic inhibitor treatment specially blocked the anti-inflammatory effect of S5 (Figs. 4, 5), suggesting that autophagy was responsible for S5 in inhibition of M1 polarization and alleviation in sepsis. Increasing researches have reported that autophagy is involved in induction and modulation of the inflammatory reaction and protects tissue from damage, and autophagy defect may lead to autoimmune diseases^35^. Beclin1, a well-known regulator for autophagy, is firstly discovered as a Bcl2 interaction protein^36^. In normal conditions, Bcl2 combinates with Bcl2 homology 3 (BH3) domain of Beclin1 and inhibits autophagy, while upon stress Beclin1 dissociates from Bcl2, allowing the activation of Vps34/Class III PI3K and subsequent stimulation of autophagy^37^. The binding of Bcl2 and Beclin1 is the best negative regulator mechanism of the autophagy function and as a novel therapeutic avenue for various diseases.^29,30^. In our studies, S5 was confirmed to dose-dependently interrupt Beclin1–Bcl2 interaction (Fig. 6a, b), which resulted in the activation of cellular autophagy and inhibition of inflammation. Meanwhile, under S5 treatment, K63-linked ubiquitination of Beclin1 was significantly increased (Fig. 6c, d). BH3 domain of Beclin1, the major site for K63-linked ubiquitination, also was the binding site for Bcl2 protein. Thus, Beclin1 ubiquitination disrupted Beclin1–Bcl2 interaction, and aggravated Beclin1–PI3KCIII interaction. As K63-linked ubiquitination of Beclin1 was increased, we subsequently focused on the upstream of ubiquitination. USP14 was reported as a DUB for Beclin1, and TRAF6 was proved to be associated with K63-linked polyubiquitination of Beclin1 (ref. ^32^). Consequently, our result showed that S5 interrupted USP14–TRAF6 interaction, and simultaneously enhanced TRAF6–Beclin1 interaction (Fig. 6e, f).

What’s more, we finally confirmed that S5 directly targeted USP14 at Ser404, Phe405, and Cys414 to inhibit its deubiquitinating enzymic activities through multiple methods (Fig. 7a–e). Therefore, it is concluded that S5 treatment directly inhibits the deubiquitinating enzymic activity of USP14, especially blocks USP14–TRAF6 interaction, and increases of TRAF6–Beclin1 association, which results in K63-linked ubiquitination of Beclin1 and autophagy. Interestingly, USP14 mutant (S404A, F405A, and C414A) moderately affected the interaction with TRAF6, compared to USP14-WT (Supplementary Fig. S6), suggesting that there may be other binding sites of TRAF6 in USP14, which is not the same as S5.

IU1 is a known USP14 inhibitor with the binding sites at His426, Tyr436, and Tyr476 probably via both hydrophobic interactions and *π*–*π* stacking^38^. IU1 inhibits USP14 by preventing its docking on the proteasome and increases K63-ubiqutination of Beclin1 (ref. ^32^) and USP14 was insensitive to IU1 in the absence of proteasomes^39^. So that, the mechanism of IU1 is distinct with S5. In our results, LC3 dots intensity achieved 100% at 6 h treatment of S5 and the EC50 value was 22.6 μM (Fig. 8g, h), while LC3 dots intensity achieved 100% at 24 h treatment of IU1 and the EC50 value was 63.4 μM (ref. ^32^). In addition, S5 induced notably K63-linked ubiquitination of Beclin1 (Fig. 7c, d), but IU1 had little effect (Fig. 8d). So, S5 was more sensitive than IU1 in autophagy induction. This was because that S5 not only inhibited deubiquitinating enzymic activity of USP14, but also greatly blocked USP14–TRAF6 interaction, which led to obvious TRAF6–Beclin1 interaction and facilitation of Beclin1 ubiquitination. Another, S5 appeared better anti-inflammatory (Fig. 8c, e, f) and less toxic effect (Fig. 8a, b) than IU1. These results prove that S5 can be served as a novel USP14 inhibitor distinguished from IU1 in autophagy induction and a prospective candidate for anti-inflammation treatment.

Sepsis begins with an explosion of inflammatory cytokines, then develops into immunosuppression phase as cytokines depletion, and eventually leads to patient death. Our results suggested that S5 targeting USP14 to enhance autophagy provides a potential therapeutic solution for sepsis. S5 showed powerful features in immunoregulation of macrophages via maintaining the balance of energy metabolism and cellular homeostasis, rather than persistent immunosuppression or killing effect which maybe aggravate sepsis.

In conclusion, the study demonstrates that S5 inhibits M1-like macrophage polarization, alleviates inflammatory responses, and provides competitively therapeutic strategy for sepsis. Regulation of USP14 offers a promising therapeutic agent for macrophage inflammatory diseases through inducing autophagy.

## Supplementary information

Supplementary Figure S1

Supplementary Figure S2

Supplementary Figure S3

Supplementary Figure S4

Supplementary Figure S5

Supplementary Figure S6

Supplementary Figure S7

Supplementary Figure Legends
