# Supplementary figures and images for: Typically inhibiting USP14 promotes autophagy in M1-like macrophages and alleviates CLP-induced sepsis
Source: Cell Death Dis. 2020 Aug 20;11(8):666. doi: 10.1038/s41419-020-02898-9 (PMC7441392; doi:10.1038/s41419-020-02898-9)

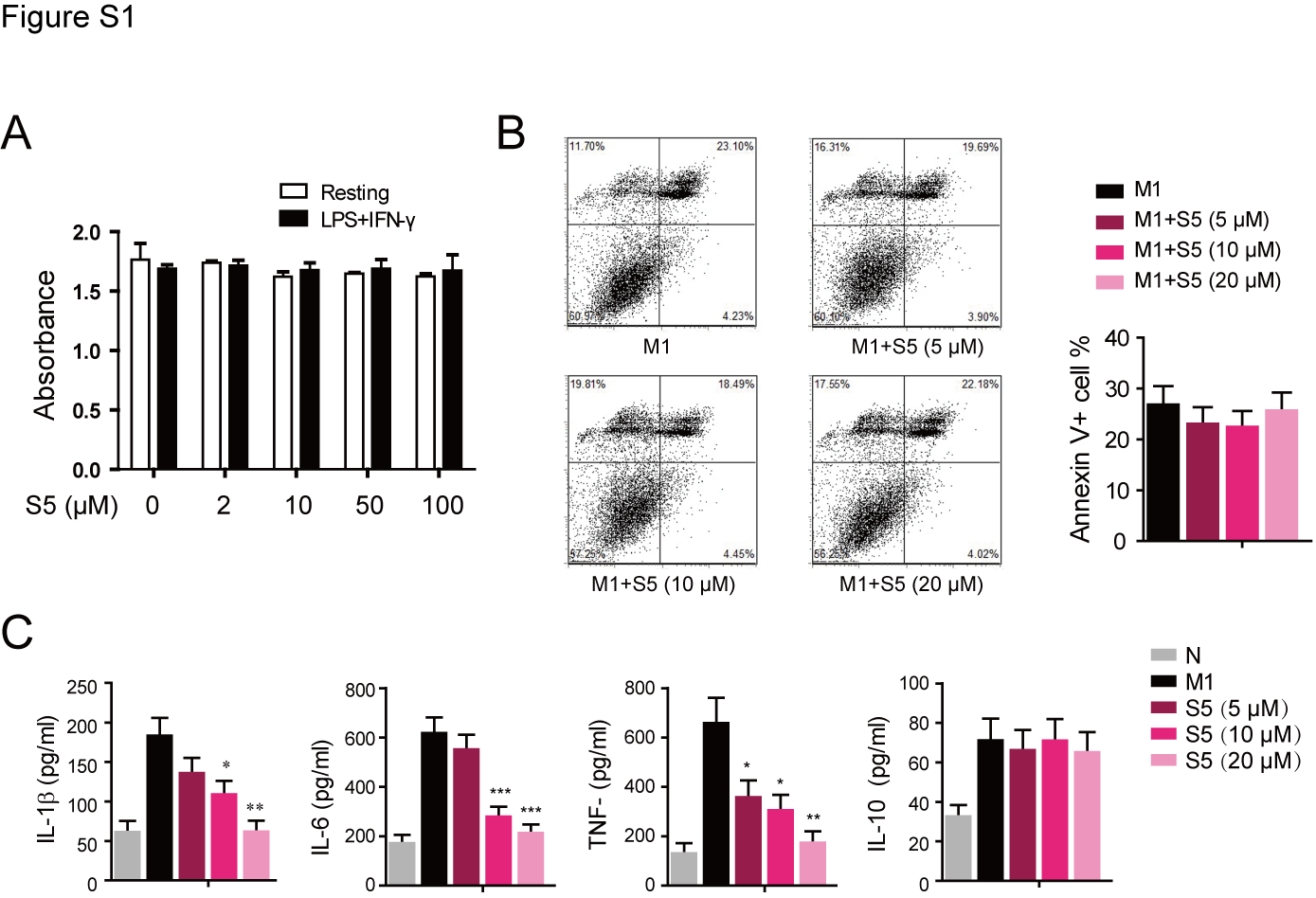

Supplement: Supplementary file 1 — Supplementary Figure S1 [file 41419_2020_2898_MOESM1_ESM.tif]

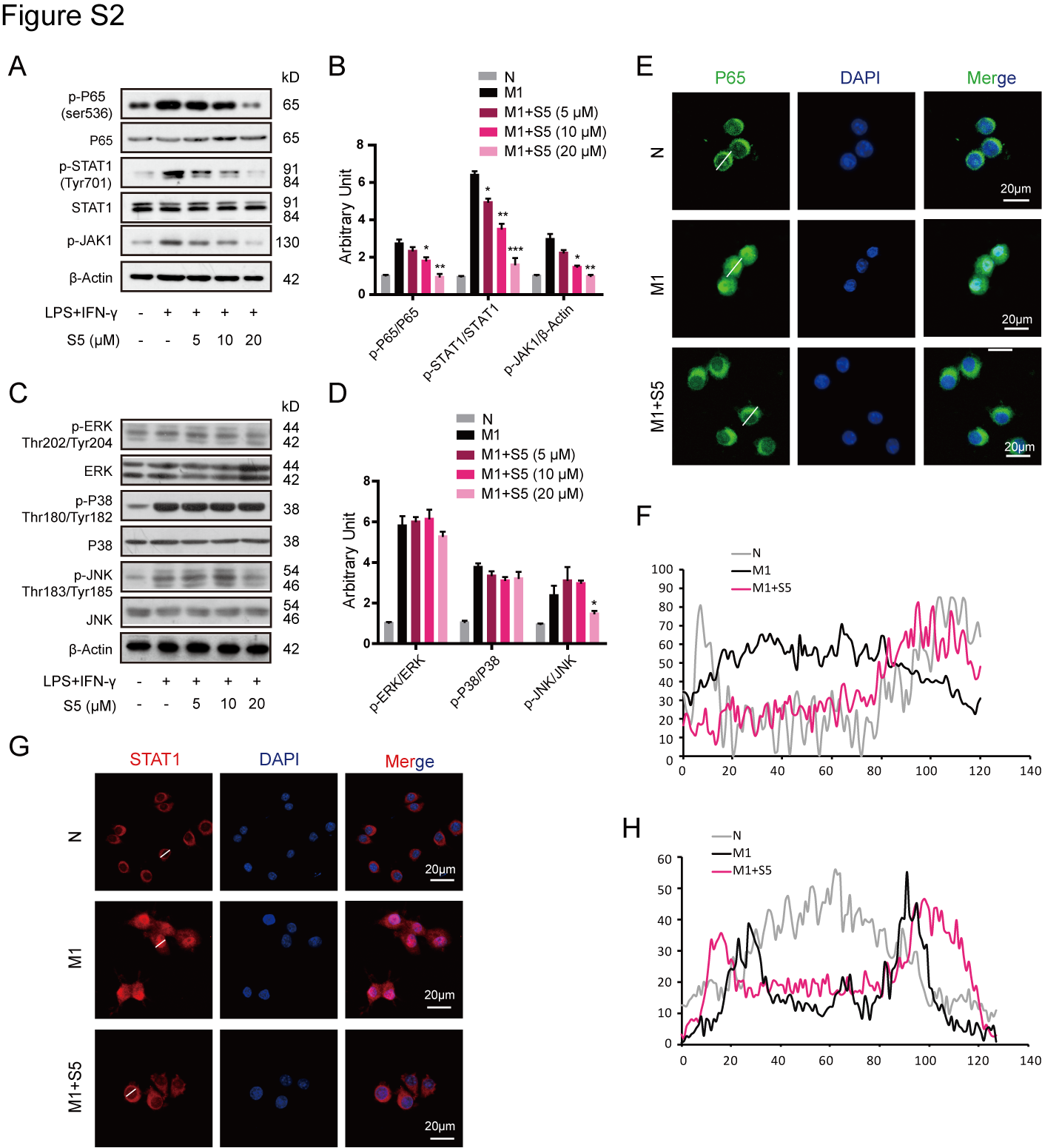

Supplement: Supplementary file 2 — Supplementary Figure S2 [file 41419_2020_2898_MOESM2_ESM.tif]

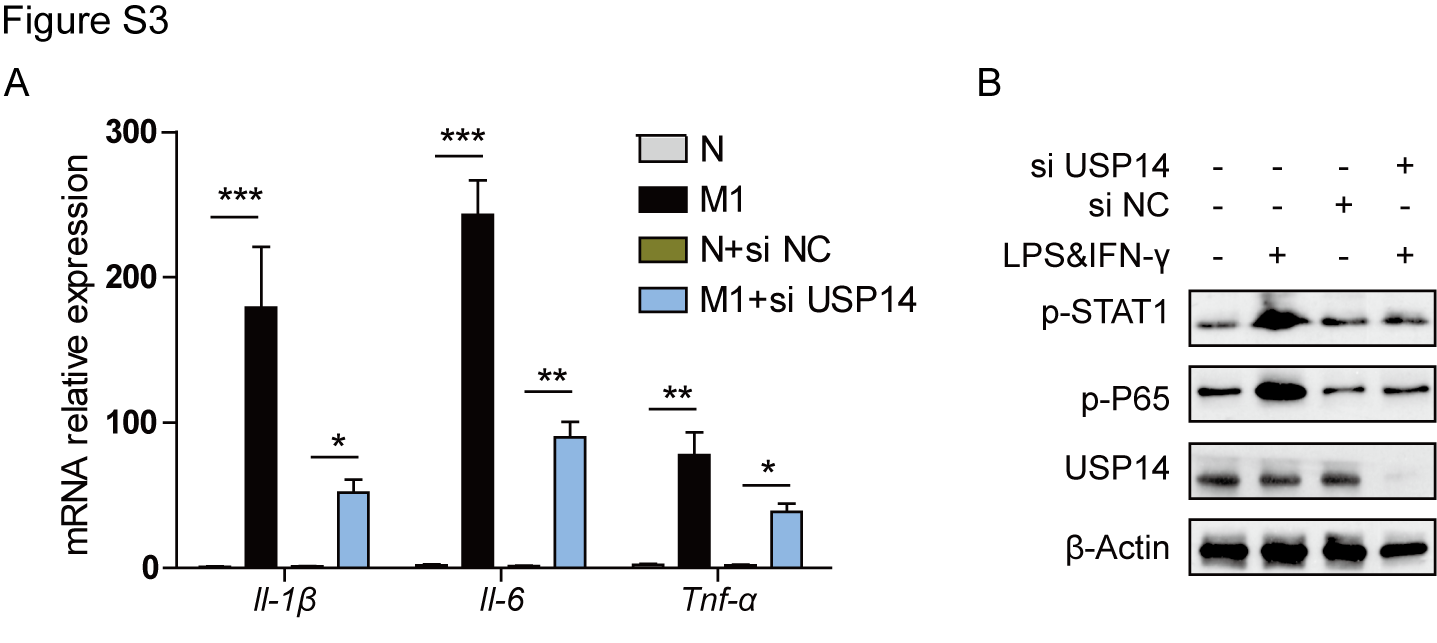

Supplement: Supplementary file 3 — Supplementary Figure S3 [file 41419_2020_2898_MOESM3_ESM.tif]

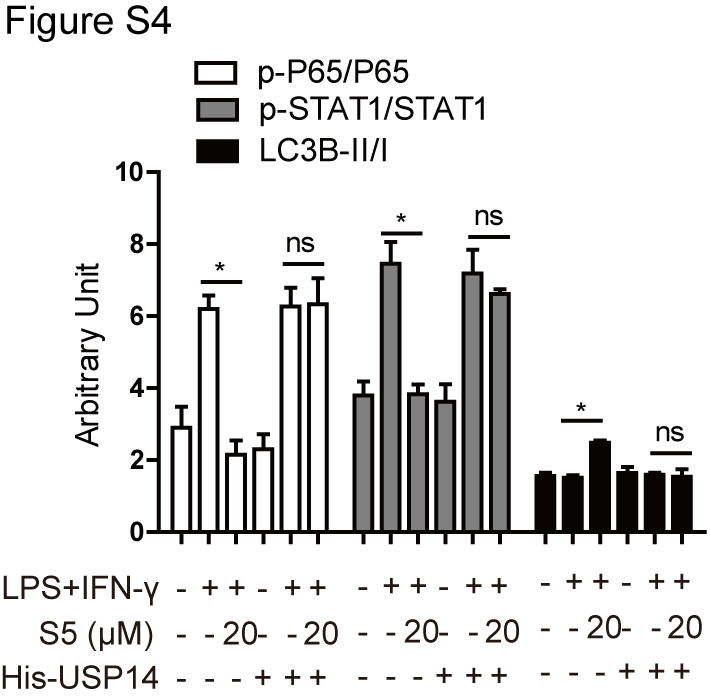

Supplement: Supplementary file 4 — Supplementary Figure S4 [file 41419_2020_2898_MOESM4_ESM.tif]

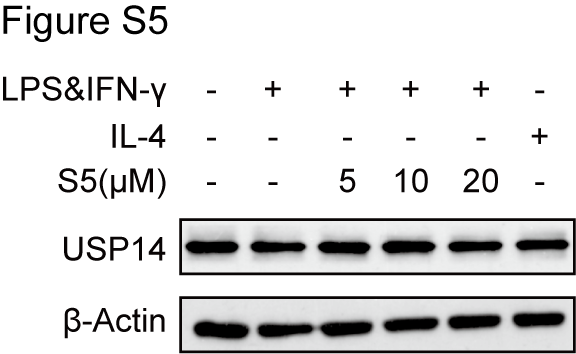

Supplement: Supplementary file 5 — Supplementary Figure S5 [file 41419_2020_2898_MOESM5_ESM.tif]

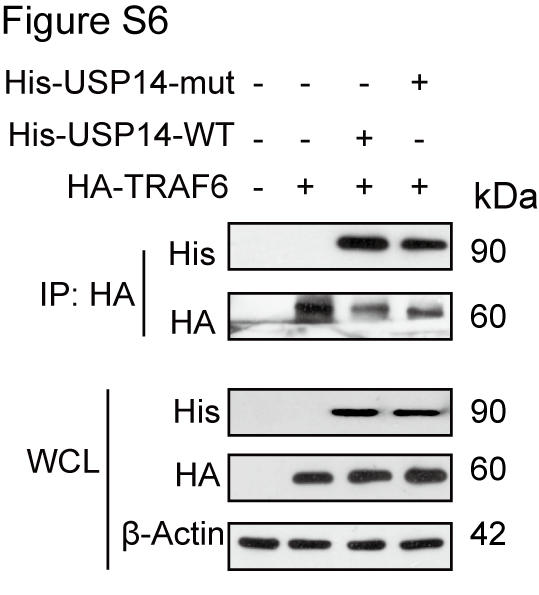

Supplement: Supplementary file 6 — Supplementary Figure S6 [file 41419_2020_2898_MOESM6_ESM.tif]

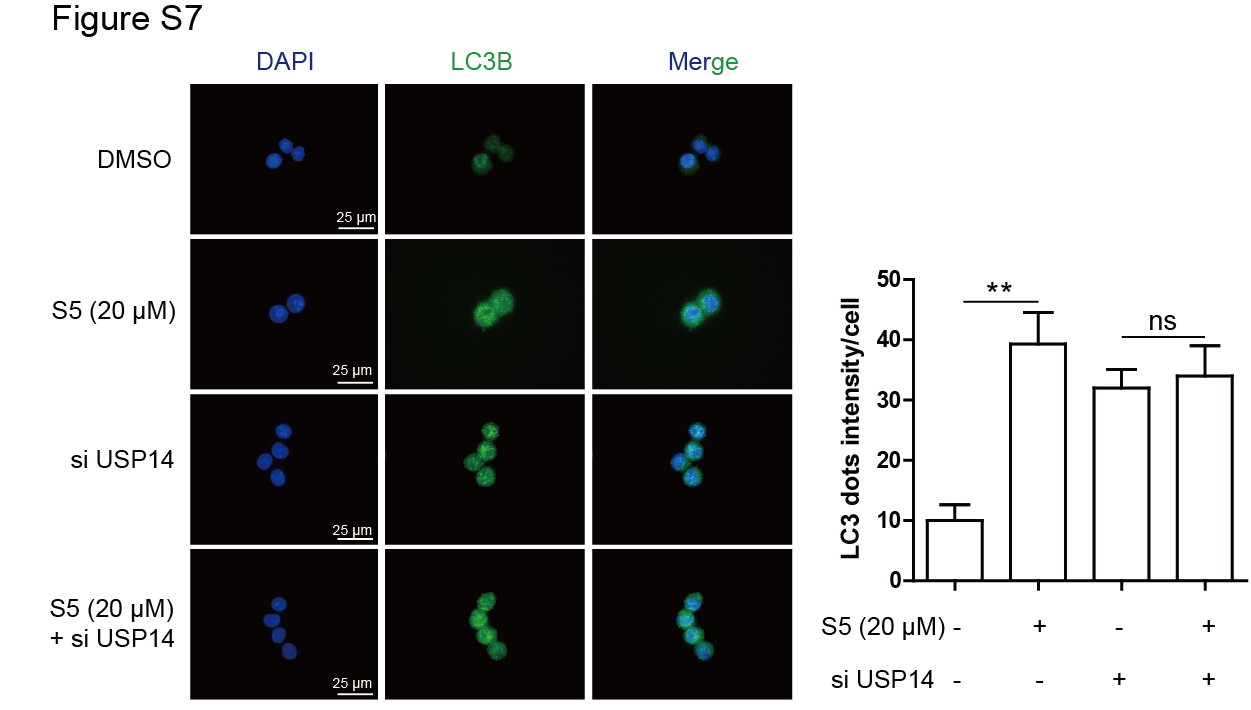

Supplement: Supplementary file 7 — Supplementary Figure S7 [file 41419_2020_2898_MOESM7_ESM.tif]
