# Supplementary material for: Typically inhibiting USP14 promotes autophagy in M1-like macrophages and alleviates CLP-induced sepsis
Source: Cell Death Dis. 2020 Aug 20;11(8):666. doi: 10.1038/s41419-020-02898-9 (PMC7441392; doi:10.1038/s41419-020-02898-9)
Supplement: Supplementary file 8 — Supplementary Figure Legends [file 41419_2020_2898_MOESM8_ESM.docx]

**Supplementary Table 1.** Primer sequences for PCR.

| Gene | Forward primer sequence (5’-3’) | Reverse primer sequence (5’-3’) |
| --- | --- | --- |
| Mouse IL-1β | CTTCAGGCAGGCAGTATCACTC | TGCAGTTGTCTAATGGGAACGT |
| Mouse IL-6 | ACAACCACGGCCTTCCCTAC | TCTCATTTCCACGATTTCCCAG |
| Mouse TNF-α | CGAGTGACAAGCCTGTAGCCC | GTCTTTGAGATCCATGCCGTTG |
| Mouse Inos | CAACATCAGGTCGGCCATCACT | ACCAGAGGCAGCACATCAAAGC |
| Mouse Fizz1 | AGGAGCTGTCATTAGGGACATC | GGATGCCAACTTTGAATAGG |
| Mouse Ym1 | AGAAGGGAGTTTCAAACCTGGT | GTCTTGCTCATGTGTGTAAGTGA |
| Mouse Cd206 | GCAGGTGGTTTATGGGATGT | GGGTTCAGGAGTGTTGTGG |
| Mouse β-Actin | TGCTGTCCCTGTATGCCTCT | TTTGATGTCACGCACGATTT |

**Supplementary Table 2.** Antibodies used for Immunofluorescence and Western Blot

| Antibody | Company | Catalogue# | RRID |
| --- | --- | --- | --- |
| Anti-P65 | Cell Signaling Technology, Beverly, MA | 8242 | AB_10859369 |
| Anti-p-P65 | Cell Signaling Technology, Beverly, MA | 3031 | AB_330559 |
| Anti-STAT1 | Cell Signaling Technology, Beverly, MA | 9172 | AB_2198300 |
| Anti-p-STAT1 | Santa Cruz Biotechnology, Santa Cruz, CA | sc-7988 | AB_656668 |
| Anti-ERK | Cell Signaling Technology, Beverly, MA | 4695 | AB_390779 |
| Anti-p-ERK | Cell Signaling Technology, Beverly, MA | 4370 | AB_2315112 |
| Anti-P38 | Cell Signaling Technology, Beverly, MA | 8690 | AB_10999090 |
| Anti-p-P38 | Cell Signaling Technology, Beverly, MA | 9216 | AB_331296 |
| Anti-JAK1 | Cell Signaling Technology, Beverly, MA | 3344 | AB_2265054 |
| Anti- JNK | Cell Signaling Technology, Beverly, MA | 9258 | AB_2141027 |
| Anti- p-JNK | Cell Signaling Technology, Beverly, MA | 4668 | AB_823588 |
| Anti- LC3B | Cell Signaling Technology, Beverly, MA | 3868 | AB_2137707 |
| Anti- P62 | Abcam, Burlingame, CA | Ab109012 | AB_2810880 |
| Anti- BECN1 | Santa Cruz Biotechnology, Santa Cruz, CA | sc-48341 | AB_626745 |
| Anti- Bcl2 | Abcam, Burlingame, CA | ab 16904 | AB_443540 |
| Anti- Flag | Cell Signaling Technology, Beverly, MA | 8146 | AB_10950495 |
| Anti- HA | Cell Signaling Technology, Beverly, MA | 2367 | AB_10691311 |
| Anti- USP14 | Cell Signaling Technology, Beverly, MA | 11931 | AB_2721157 |
| Anti- PI3KCIII | Cell Signaling Technology, Beverly, MA | 4263 | AB_2299765 |
| Anti-β-Actin | Cell Signaling Technology, Beverly, MA | 58169 | AB_2750839 |
| Anti- TRAF6 | Santa Cruz Biotechnology, Santa Cruz, CA | Sc-8409 | AB_628391 |
| CD11c-FITC | Biolegend (San Diego, CA) | 117306 | AB_313775 |
| CD206-APC | Biolegend (San Diego, CA) | 141707 | AB_10896057 |
| CD11b-PE | ThermoFisher Scientific, Waltham, MA | 12-0112-81 | AB_465546 |
| F4/80 PE-Cyanine7 | ThermoFisher Scientific, Waltham, MA | 25-4801-82 | AB_469653 |
| Alexa Fluor® 647  anti-mouse Ly-6C | Biolegend (San Diego, CA) | 128009 | AB_1236551 |
| Alexa Fluor® 488  anti-mouse Ly-6G | Biolegend (San Diego, CA) | 127625 | AB_2561339 |
| Alexa Fluor 488  Goat anti-Rabbit IG | ThermoFisher Scientific, Waltham, MA | A-11034 | AB_2576217 |
| Alexa Fluor 594  Goat anti-Rabbit IG | ThermoFisher Scientific, Waltham, MA | A-11037 | AB_2534095 |

**Supplementary Fig. S1** **Cell viability test of S5 in BMDMs.** (A). BMDM cells were incubated with various concentrations of S5 or the same volume of DMSO in the absence or presence of 10 ng/ml LPS & IFN-γ for 24 h. Cell proliferation was detected by MTT assay. (B). Cells were stained with Annexin V/PI and assayed by flow cytometry. (C). Cytokines level of IL-1β, IL-6, TNF-α, IL-10 secreted in M1-RAW 264.7 cells were assessed by ELISA. Data are means ± SEM of five independent experiments. *P < 0.05 significantly different from LPS&IFN-γ group.

**Supplementary Fig. S2. S5 regulated macrophage polarization pathway.** (A and C). BMDMs were activated by 10 ng/ml LPS and 10 ng/ml IFN-γ and incubated with 5 μM, 10 μM, or 20 μM S5 or the same volume of DMSO, protein expression was analyzed by immunoblotting. S5 inhibited phosphorylation level of P65, STAT1 and JAK1 (A) and had no effect on p-ERK, p-p38, and p-JNK (C). (B and D). Data summary were expressed as a histogram of mean±SEM of five independent experiments. *P < 0.05 significantly different from LPS & IFN-γ group. (E and G). Cytoplasmic nuclear localization of P65 and STAT1 were analyzed by immunofluorescence staining. S5 suppressed p65 (E) and STAT1 (G) nuclear import. (F and H). The line charts represent the mean fluorescence intensity (MFI), Scale bar, 25 μm.

**Supplementary Fig. S3. USP14 knockdown inhibited M1-like macrophage polarization.** USP14 knockdown in RAW 264.7 cells reduced *Il-1β*, *Il-6* and *Tnf-α* mRNA level (A) and depressed p-P65 and p-STAT1 expression (B) compared to LPS&IFN-γ treatment.

**Supplementary Fig. S4. USP14 overexpression partially weakened effect of S5.** This is the statistics for Fig. 6H.

**Supplementary Fig. S5. USP14 expression in M1-like and M2-like macrophages.** Protein level of USP14 in M1 and M2 macrophages had no difference, and S5 did not affect it.

**Supplementary Fig. S6. USP14 and TRAF6 Interaction assay.** USP14-mutants (S404A, F405A, C414A) moderately suppressed the interaction with TRAF6, compared to USP14-WT.

**Supplementary Fig. S7. USP14 knockdown greatly blocked the effect of S5 in autophagy.** RAW264.7 cells were transfected with si USP14 or si Control for 48h, and LC3B expression was analyzed by immunofluorescence staining with or without S5 treatment. Scale bar, 25 μm. The average LC3 dots intensity in 1000 cells from each indicated sample was determined. Data are means ± SEM of three independent experiments. ** P<0.01 vs. DMSO group.
